# Dioscin inhibits non-small cell lung cancer cells and activates apoptosis by downregulation of Survivin

**DOI:** 10.7150/jca.89831

**Published:** 2024-01-20

**Authors:** Ruirui Wang, Xiaoying Li, Yu Gan, Jinzhuang Liao, Shuangze Han, Wei Li, Gaoyan Deng

**Affiliations:** 1 Department of Thoracic Surgery, Hunan Chest Hospital, Changsha 410013, Hunan, China.; 2 Department of Radiology, The Third Xiangya Hospital of Central South University, Changsha, Hunan 410013, China.; 3 Department of Ultrasound, Union Hospital, Tongji Medical College, Huazhong University of Science and Technology, Wuhan, Hubei 430022, China.

**Keywords:** NSCLC, Survivin, Dioscin, Ubiquitination

## Abstract

Human malignancies exhibit elevated levels of survivin, and have been linked to poor prognosis. Targeting survivin expression is a promising therapeutic strategy against cancer cells. Natural compounds have become a hot topic in research due to their non-toxic, non-invasive, and efficient treatment of multiple diseases. In this current investigation, it was discovered that Dioscin, as a natural compound, exerted profound antitumor activity against NSCLC cell lines, inhibiting NSCLC cell viability and promoting apoptosis. Further mechanistic studies showed that Dioscin promoted ubiquitination-mediated survivin degradation via strengthening the interaction between survivin and the E3 ubiquitin ligase Fbxl7. Furthermore, Dioscin exhibited a strong tumor suppressive effect in xenograft tumor models, and Dioscin treatment led to a notable decrease in tumor volume and weight. Based on our findings, Dioscin is expected to be a potential antitumor agent for non-small cell lung cancer treatment.

## Introduction

Lung cancer is the most common cause of cancer deaths worldwide, with reports indicating that approximately 1.8 million people die from lung cancer each year 1. Approximately 85% of lung cancers are Non-small-cell lung cancer (NSCLC), whose incidence increases yearly 2. While numerous studies have demonstrated that smoking constitutes the foremost risk factor in developing NSCLC cancer, other risk factors include genetic history, toxic substance exposure, etc. 3, the mechanism of NSCLC tumorigenesis is not yet clear. According to the stage of lung cancer, histology, genetic alterations, and individual patient differences, the treatments for NSCLC generally include radiotherapy, surgery, immunotherapy, chemotherapy, and molecular targeted therapy. In recent years, we have made remarkable strides in targeted therapy and immunotherapy for NSCLC 3, 4. However, early diagnosis of NSCLC, control of metastatic spread, and prevention of therapeutic resistance remain the main challenges to control and eradicate the disease 5. Therefore, elaboration of the pathogenesis of NSCLC and the discovery of new antitumor targets and therapeutic approaches remains major obstacles to be solved.

Survivin, belonging to the family of inhibitory apoptosis proteins (IAPs), stands out as one of the most significant oncogene proteins identified up to this point 6. It exhibits high expression levels in various tumor tissues, such as lung cancer, pancreatic cancer, breast cancer, colorectal cancer (CRC), and liver cancer, and exerts a key role in proliferation, apoptosis, angiogenesis, and therapy resistance 7-14. Survivin can bind to caspases to inhibit apoptosis, regulate microkinetics in the G2/M phase of proliferating cells to promote mitosis, and form chromosome passenger complexes (CPC) with CDCA8/Borealin, AURKB (Aurora B kinase) and INCENP (inner centromere protein) 15. Recent studies have shown that survivin could be a biomarker and potential prognostic indicator for breast cancer (BC) 16, 17. Survivin overexpression in head and neck squamous carcinoma cells (HNSCC) exhibits treatment resistance 18, 19. Targeting survivin can make cervical cancer cells more sensitive to radiation therapy 20. Thus, survivin plays a critical regulatory role in both apoptosis and mitosis. Consequently, all these biological characteristics endow survivin as an attractive and promising therapeutic target against cancer cells 21-23.

Dioscin is a natural plant-derived steroidal saponin, which has a good curative effect on metabolic disorders, organ damage, viral and fungal infections, and multiple malignant tumor cells, and it has gained immense attention and popularity in preclinical research 24. Notably, Dioscin was found to suppress tumorigenesis of colitis-associated CRC by modulating myeloid-derived suppressor cells (MDSCs) and macrophage polarization 25. Additionally, Dioscin has demonstrated the capability to inhibit osteosarcoma stem-cell-like characteristics and tumor growth by repressing the Akt/GSK3/β-catenin signaling pathway 26. Accumulating studies have elucidated the potent antitumor pharmacological activity of Dioscin in human cancers, which can be attributed to DNA damage, apoptosis, the induction of cell cycle arrest, and reprogramming glycolysis 24, 27, 28. However, the potential antitumor mechanism of Dioscin on NSCLC has not been comprehensively clarified. Thus, the primary objective of this study is to explore the pharmacological activity of Dioscin on NSCLC cells and delve into the underlying mechanism responsible for its antitumor effect.

## Materials and methods

### Chemical reagents and cell culture

293T, human NSCLC cell lines containing H1975, HCC827, and A549, and normal non-tumor cells HBE, these cell lines were obtained from the American Type Culture Collection (ATCC, Manassas, VA). Following ATCC protocols, all cells were maintained in a humidified incubator (37°C, 5% CO_2_) and underwent mycoplasma analysis every two months. Chemicals, incorporating SDS, NaCl, Tris base, DMSO, and lipofectamine™ 2000 (#11668019), were gained through Sigma-Aldrich (St. Louis, MO). Dioscin, cycloheximide (CHX) and MG132 were sourced from Selleck Chemicals (Houston, TX). The RPMI-1640 and DMEM culture media, along with Fetal Bovine Serum (FBS) and penicillin-streptomycin (P/S), were sourced from Invitrogen (Grand Island, NY) to support the cell culture process. Primary antibodies targeting ki67 (#ab16667, IHC: 1:300) and FbxL7 (#ab59149, IB: 1:1000) were obtained from Abcam (Cambridge, United Kingdom) for use in this study, cytochrome C (#11940, IB: 1:1000), survivin (#2808, IB: 1:1000, IP: 1:200, IHC: 1:200), ubiquitin (#3936, IB: 1:1000), β-actin (#3700, IB: 1:10000), α-tubulin (#3873, IB: 1:5000), VDAC1 (#4661, IB: 1:2000), Bax (#14796, IB: 1:1000), cleaved-caspase 3 (#9664, IB: 1:1000, IF: 1:400) were obtained from Cell Signaling Technology, Inc. (Beverly, MA). We obtained the Flag-survivin construct from Origene (RC205935).

### MTS assays

After counting, NSCLC cells were plated in 96-well plates with a density of 3×10^3^ cells per well and cultured overnight. Cells were exposed to various doses of Dioscin for 24, 48, and 72 hours. Finally, cell viability was assessed following standard protocols using MTS reagent (#G3580, Madison, WI).

### Anchorage-independent cell growth

The methodology for the Anchorage-independent cell growth assay was executed following the previously established protocol 29. Briefly, NSCLC cells (8×10^3^) were cultured in 6-well plates containing 0.6% Basal Medium Eagle agar supplemented with 10% FBS and different concentrations of Dioscin. Subsequently, the cells were cultured under controlled conditions at 37°C with 5% CO_2_ for 14 days to support their growth.

### Immunofluorescence (IF)

The immunofluorescence staining was conducted as previously described 30. Cells were placed on chamber slides and treated with 4% paraformaldehyde for 20 minutes. After permeabilization in 0.5% Triton X-100 for an additional 20 min, cells were incubated in 5% BSA for 1 h. Afterward, the cells were exposed to cleaved-caspase 3 antibodies and incubated overnight at 4°C. Afterward, the cells underwent a PBS wash and were incubated with Alexa Fluor 568 dye-labeled anti-rabbit IgG. Finally, DAPI (P36935, Thermo Fisher Scientific) was used for nuclear staining for 30 min. Fluorescence images were acquired utilizing the confocal microscope system (Nikon C1si; NIKON Instruments Co.).

### Flow cytometry

The procedure for apoptosis analysis using flow cytometry was carried out following the previously established method 31. In summary, NSCLC cells underwent a 24-hour exposure to Dioscin. Subsequently, 1×10^6^ cells were suspended in 500 μl of binding buffer, followed by the addition of 5 μl of Annexin V-FITC and 10 μl of PI, mixed thoroughly, and incubated for 5 minutes at room temperature and protected from light. Finally, the apoptotic cells were analyzed by FACS.

### Western blotting (WB)

WB was implemented as formerly depicted 32. Shortly, Whole-cell extract (WCE) was formulated by RIPA buffer supplemented with proteinase inhibitors and concentrated through BCA protein evaluation. The entire 20 μg WCE blended with loading buffer seethed at 95 °C for 5 min. Following this, the next steps included SDS-PAGE electrophoresis and electroblotting. Seal the membranes with 5% non-fat milk for 1 hour at room temperature, then incubate the corresponding antibody at 4 °C overnight. Subsequently, membranes were treated with secondary antibodies for 1 h at ambient temperature. The interest protein was discernible by chemiluminescence for immunoblot (IB) analysis.

### Ubiquitination analysis

The Ubiquitination examination was implemented as formerly depicted 33. NSCLC cells were treated with Dioscin and lysed with modified RIPA buffer. The cell lysates were sonicated and heated to 95°C for 15 min, then were mixed with RIPA buffer containing 0.1% SDS for dilution and centrifuged at 16000×g for 15min. The supernatants were incubated overnight with survivin antibody conjugated protein A-Sepharose beads at 4 °C with rotation. Subsequently, the beads underwent a washing process and boiled in 2×loading buffer for 5 min. The protein supernatant was collected after centrifugation at 3000 rpm for 1 min, and then subjected to western blotting assay. The ubiquitination level of survivin was detected by incubating with ubiquitin (Ub) antibody.

### Co-immunoprecipitation (Co-IP) assays

We conducted Co-IP assays following previously established methods 34. Cells were cultured in the medium of RPMI 1640 and 10% FBS and treated with Dioscin or DMSO control for 24 hours. Cells were reaped in IP lysis buffer (#87788, Thermo Scientific) as same as the manufacturer's manual. Following the guidelines provided by the manufacturer, we assessed the protein concentration using the BCA protein assay kit. Protein A/G-agarose beads were pre-cleared and immunoprecipitated with 2 μg of survivin antibody at 4°C overnight. The next day, the cell lysates were left to incubate with the above beads at 4°C for 2 hours. SDS-PAGE separated immunocomplexes and co-immunoprecipitated proteins were measured by using related antibodies.

### Immunohistochemical (IHC) Staining

Tumor tissues were collected, fixed, and subjected to IHC analysis following established protocols 35. The tissue sections underwent a process of deparaffinization using xylene, rehydrated with ethanol, and then placed in 10 mM citrate buffer and boiled for 10 min to achieve antigen retrieval. Following washing with ddH_2_O three times, the slides underwent treatment with a 3% H_2_O_2_ solution in methanol for 10 minutes to deactivate peroxidase activity. Followed by blocking with 50% goat serum albumin in PBS at ambient temperature for 1 h, the slides underwent an overnight primary antibody incubation at 4°C. Then, tissue slides were washed thrice with PBS and treated with a secondary antibody for 45 min at 37°C. Finally, positive staining was observed using the 3,3'-diaminobenzidine (DAB) substrate. Hematoxylin was applied as a counterstain.

### Subcellular Fraction Isolation

A549 cells were treated with Dioscin for 24 hours. According to the standard instructions of the Kit, subcellular Protein Fractionation Kit (Thermo Fisher Scientific, Inc. #78840) and Mitochondria Isolation Kit (Thermo Fisher Scientific, Inc. #89874) were used to prepare proteins from different cellular compartments.

### Cycloheximide assay

Dioscin treatment was applied to HCC827 cells for 24 hours, and CHX was added to the culture medium at a final concentration of 20 ug/ml. Cell lysates were collected at 0, 2, 4, and 8 h after CHX treatment. The half-life of survivin was detected by WB assay.

### siRNA transfection

A549 cells were seeded in 6-well plates and transfected with FBXL7 siRNA (sc-62306, Santa Cruz Biotechnology, Inc.) and Ctrl siRNA (sc-37007, Santa Cruz Biotechnology, Inc.) using HiPerFect transfection reagent (Cat. 301705, Qiagen, Inc.) following the Kit protocols. Cells were incubated with siRNA transfection reagent for 48 h, and WB assay was then performed to confirm that FBXL7 was knocked down.

### Blood assay

Blood was gathered from mice through a heart prick using EDTA-coated tubes. Red blood cell (RBC) and white blood cell (WBC) counts, along with alanine aminotransferase (ALT) and aspartate aminotransferase (AST) levels, were analyzed at the laboratory of Central South University, China.

### *In vivo* tumorigenicity assessment

The maintenance and experimentation of all mice were authorized by the Institutional Animal Care and Use Committee (IACUC) of Central South University (Changsha, China). Briefly, A549 (2×10^6^) cells were s.c.inoculated onto the right side of 6-week-old female thymus-free nude mice to create the xenograft mouse model. Tumor volume and mouse body weight were detected every two days. Upon the tumor volume attaining 100 mm^3^, the mice with established tumors were randomly divided into two cohorts (n=6). The compound-treated group was given an intraperitoneal injection of Dioscin dissolved in 70% corn oil+30% PEG400 (10 mg/kg every 2 days), and the control group received the vehicle control treatment. Tumor volume was determined according to the following formula: length×width×width/2. Upon reaching the experimental endpoint, mice were euthanized, and the xenografts were removed, weighed, and then processed for immunohistochemical analysis.

### Statistics assay

GraphPad Prism 5 was used for statistical analysis of tentative data. The discrepancy between the test groups was appraised by one-way ANOVA or t-test, and the statistical meaning benchmark was p under 0.05. All mensurable experimental data exhibited qua mean ± sd.

## Results

### Dioscin inhibits NSCLC cells *in vitro*

**Figure 1:**
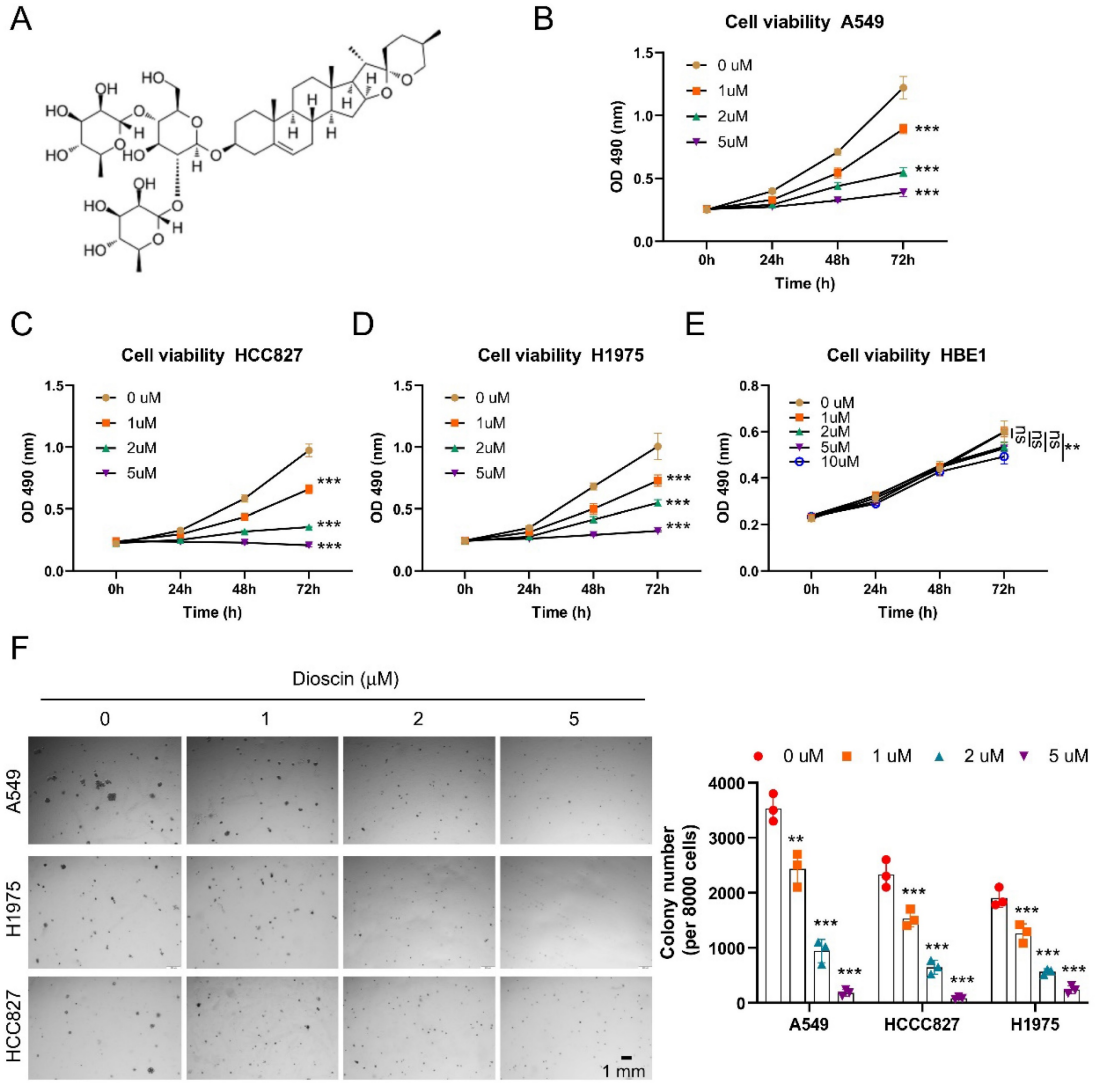
Dioscin inhibits NSCLC cells *in vitro*. (A)The molecular configuration of Dioscin. (B-D) Assessment of cellular viability in A549 (B), HCC827 (C), and H1975 (D) cell lines following exposure to Dioscin treatment through MTS assay. ***p<0.001. (E) MTS assay analysis of the cell viability of HBE1. **p<0.01. (F) Analysis of colony formation in A549, HCC827, and H1975 cells using an anchorage-independent cell growth assay. Scale bar, 1 mm.**p<0.01, ***p<0.001. ns, not statistically significant.

In preclinical research, the remarkable therapeutic effect of the natural product Dioscin (Fig. 1A) makes it a potential antitumor agent 24. Here, our findings demonstrated that Dioscin exerted a dose-dependent inhibitory impact on the cell viability of A549, HCC827, and H1975 cells (Fig. 1B-D). After 5 μM Dioscin treatment for 72 h, the viability of all three groups of NSCLC cells decreased by more than 90%. Notably, the results indicated that the cell viability of immortalized non-tumor cells HBE1 was not notably affected following treatment with Dioscin (Fig. 1E). Next, the anchorage-independent cell growth assay was performed to detect the anti-tumor activity of Dioscin, and findings pointed out that the formation of colonies of NSCLC cells in soft agar was significantly hindered after exposure to Dioscin. And the inhibitory effect of Dioscin on the aforementioned three tumor cells was enhanced in a dosage-related manner (Fig. 1F). These data above suggest that Dioscin exerts robustly antitumor activity against NSCLC cells *in vitro*.

### Dioscin induces apoptosis in NSCLC cells

**Figure 2:**
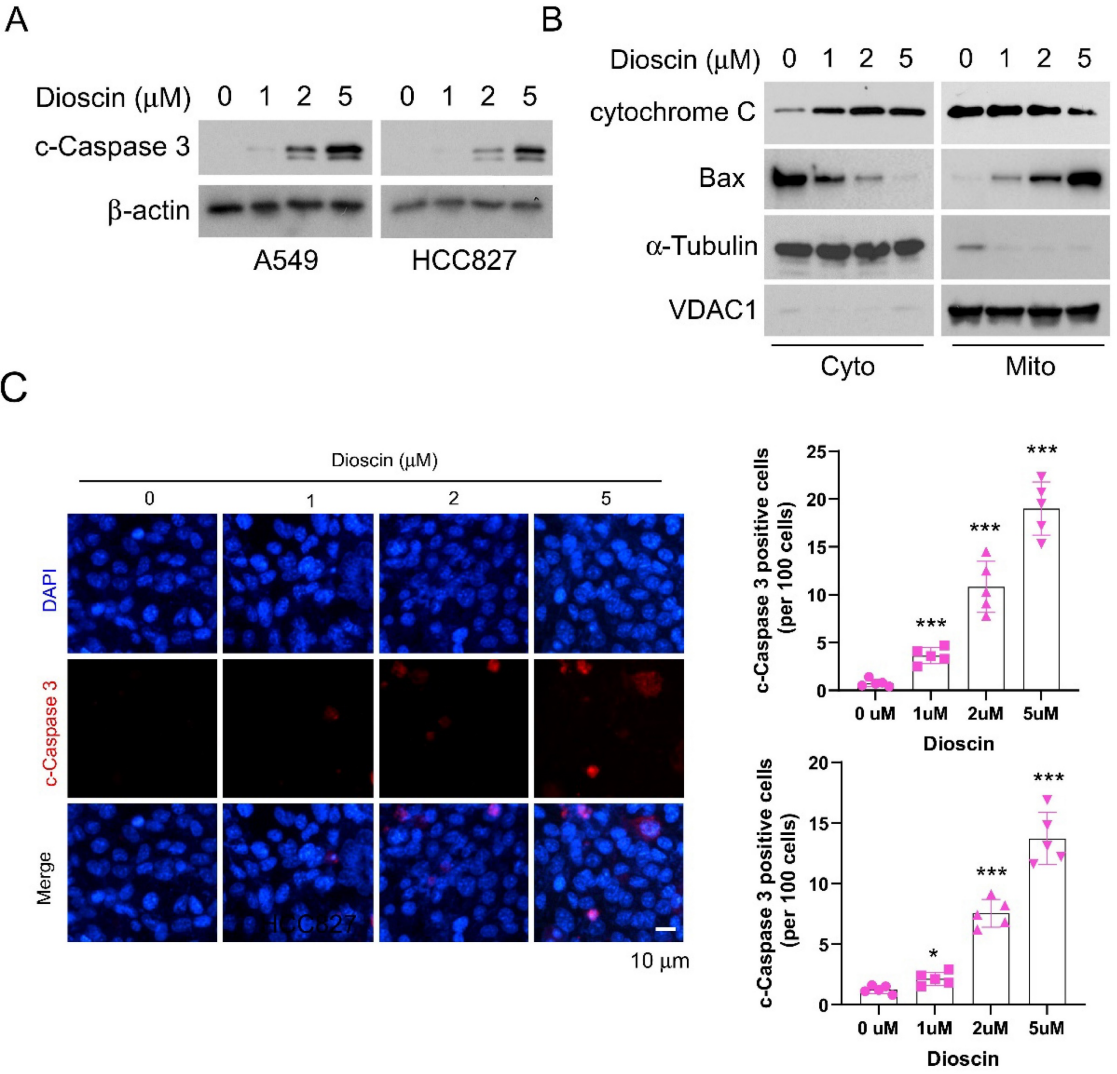
Dioscin induces apoptosis in NSCLC cells. (A) A549 and HCC827 cells were exposed to varying concentrations of Dioscin for 24 hours. Subsequently, the whole-cell extract (WCE) was utilized for immunoblotting analysis. (B) Dioscin treatment was administered to A549 cells for 24 hours, followed by the isolation of subcellular fractions and subsequent immunoblotting analysis. (C) Immunofluorescence (IF) analysis of c-Caspase 3 in HCC827 cells and A549 cells subjected to varying concentrations of Dioscin treatment. Scale bar, 10 µm. ***p<0.001.

**Figure 3:**
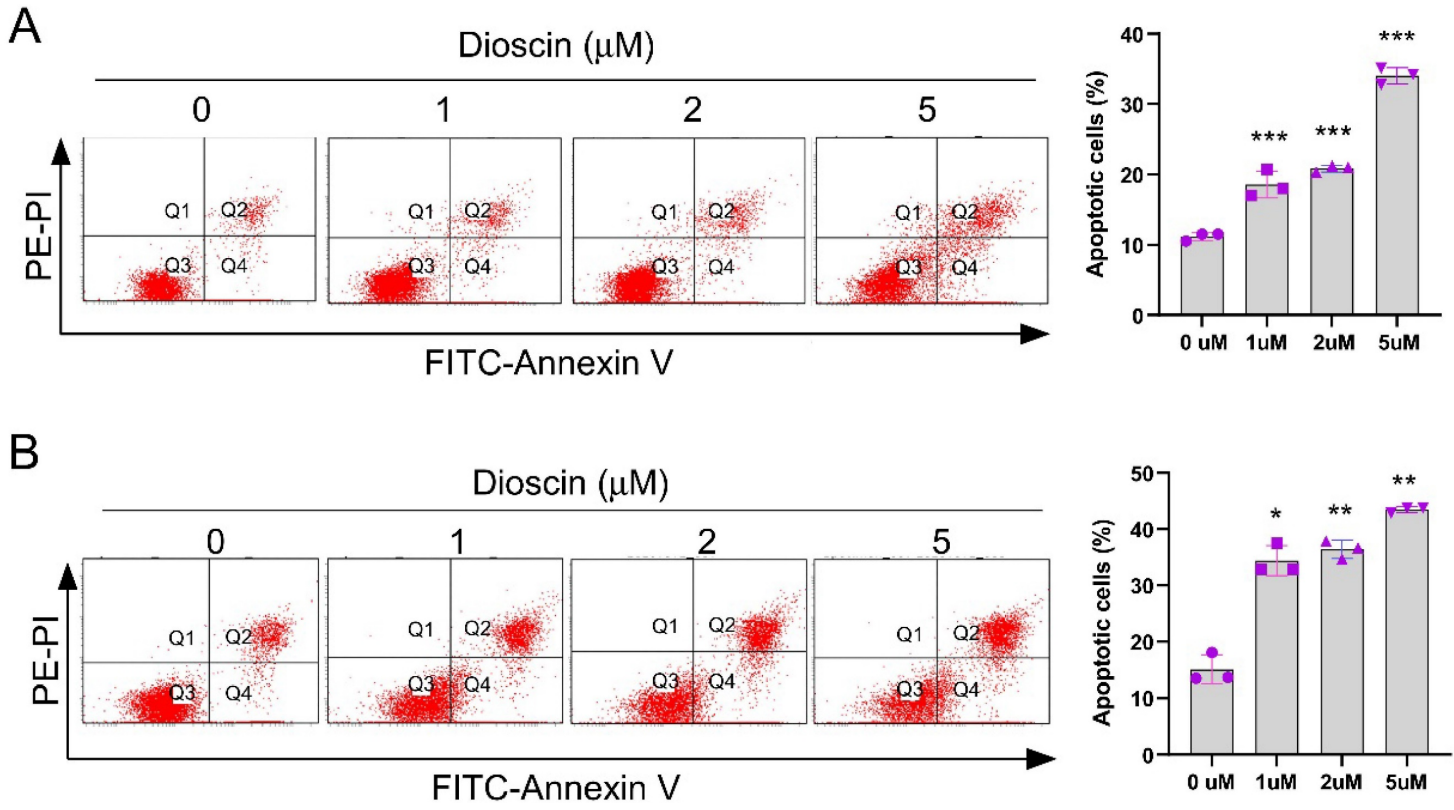
Dioscin promotes apoptosis *in vitro*. (A and B) Detection of apoptosis in A549 cells and HCC827 cells treated with different concentrations of Dioscin by flow cytometry. ***p<0.001.

It has been verified that Dioscin inhibited tumor growth and is partly attributed to induce apoptosis 36. We then examined whether apoptosis signaling was activated in NSCLC cells following treatment with Dioscin. As shown in Fig 2A, we found that the protein expression of cleaved-Caspase 3 (c-Caspase 3) increased in A549 and HCC827 cells after treatment with Dioscin (Fig. 2A). Next, we extracted subcellular fractions from Dioscin-treated A549 cells. The findings indicated an elevation in the migration of cytochrome C from mitochondria to the cytoplasm, along with an increase in Bax protein levels within mitochondria, as the concentration of Dioscin rose (Fig. 2B). Moreover, IF results showed that Dioscin treatment promoted apoptosis in HCC827 and A549 cells, as the number of c-Caspase 3 positive cells increased substantially (Fig. 2C). We next conducted Annexin V/PI staining to measure the effect of Dioscin on the induction of apoptosis in A549 and HCC827 cells. The results demonstrate that the application of Dioscin for 24 h triggered significant apoptosis dose-dependently (Fig. 3A and 3B).

### Dioscin promotes survivin degradation in NSCLC cells

**Figure 4:**
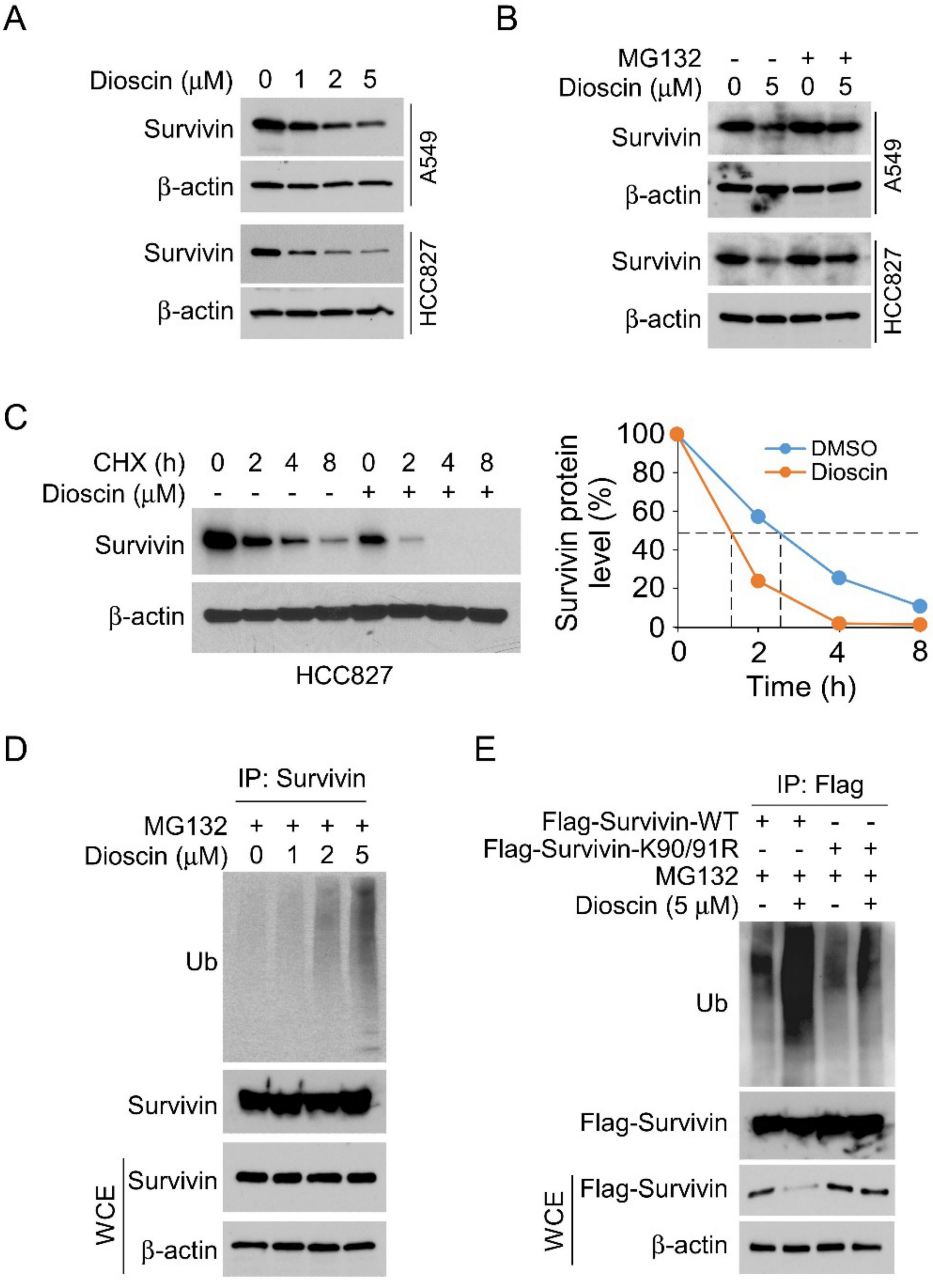
Dioscin promotes survivin degradation in NSCLC cells. (A) A549 and HCC827 cells were treated with Dioscin for 24 h. The WCE underwent IB analysis. (B) Dioscin treatment was administered to A549 and HCC827 cells for 24 hours, followed by an 8-hour incubation with MG132 (20 µM). The WCE was subjected to IB analysis. (C) Dioscin treatment was applied to HCC827 cells for 24 hours, and subsequently, cells were exposed to various time points of CHX incubation. The whole-cell extract (WCE) was then used for immunoblotting analysis. (D) HCC827 cells were treated with Dioscin for 24 h, and incubated with MG132 (20 µM) for 8 h. The WCE underwent analysis for survivin ubiquitination. (E) Following transfection with different constructs for 48 hours, HCC827 cells were exposed to Dioscin treatment for 24 hours and subsequently the cells were subjected to an 8-hour incubation with MG132 (20 µM). Survivin ubiquitination analysis was performed on the whole cell extract (WCE).

To further investigate the underlying mechanism of Dioscin-induced apoptosis in NSCLC cells, we first examined survivin, one of the most critical anti-apoptotic proteins. Interestingly, IB results demonstrated that the expression level of survivin decreased with increasing Dioscin concentration in A549 and HCC827 cells (Fig. 4A). Furthermore, the protein level of survivin was restored following treatment with MG132, a proteasome inhibitor, even in the presence of Dioscin in NSCLC cells (Fig. 4B). As depicted in Fig. 4C, the results manifested that the half-life of survivin was obviously reduced in HCC827 cells treated with Dioscin, suggesting that Dioscin-induced survivin downregulation was related to its stability change. Subsequently, the ubiquitination level of survivin was determined. The results indicated that the ubiquitination of survivin was dose-dependently increased by Dioscin treatment in HCC827 cells (Fig. 4D). Moreover, our data indicated a mutation at K90/91, which has a pivotal function in maintaining the stability of survivin^37^, reduced Diosicn-induced survivin ubiquitination in HCC827 cells (Fig. 4E). These results illustrated that Dioscin induced-survivin ubiquitination and degradation might be associated with apoptosis activation.

### Fbxl7-mediated ubiquitination is required for Dioscin-induced survivin degradation

**Figure 5:**
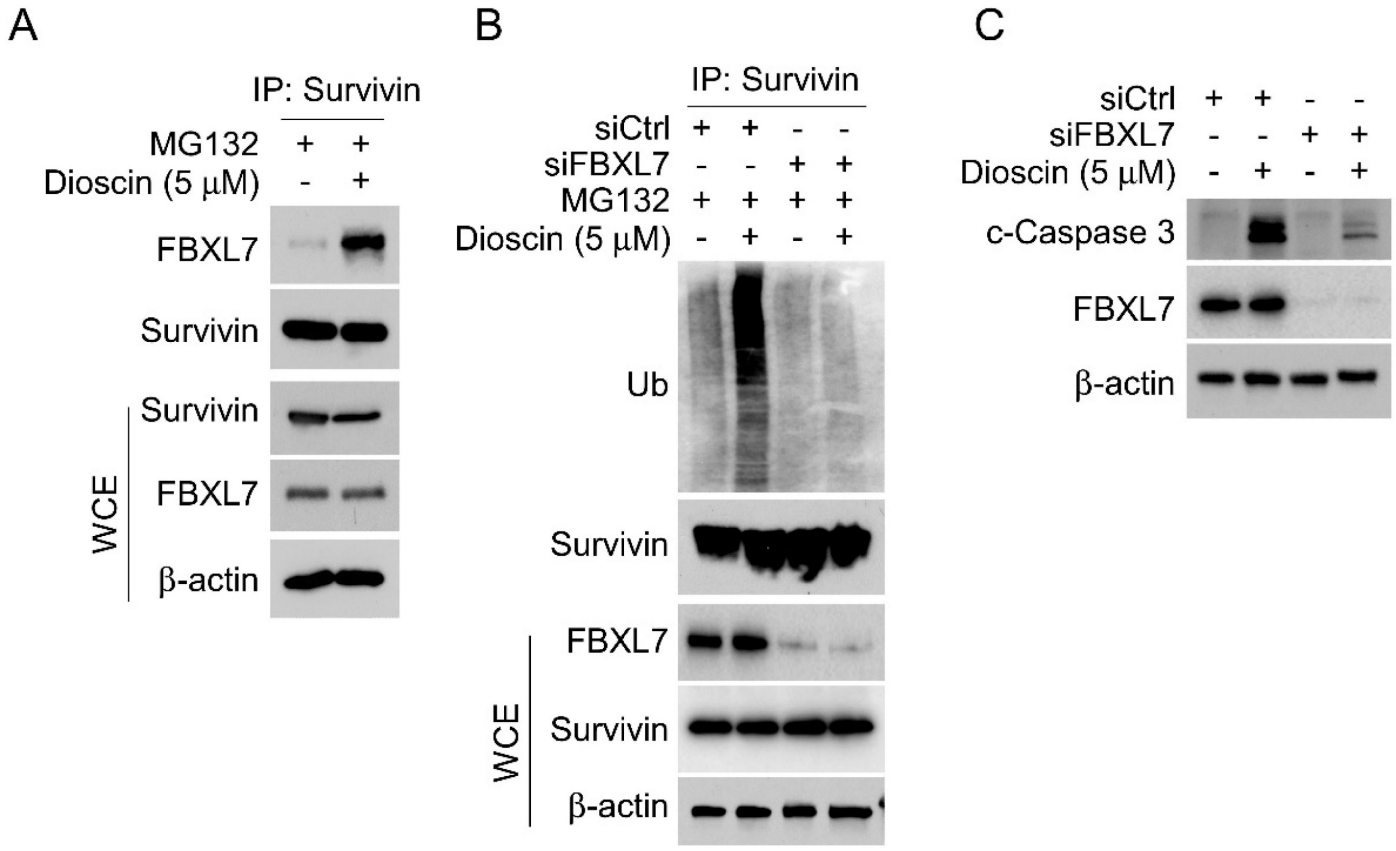
Fbxl7-mediated ubiquitination is required for Dioscin-induced survivin degradation. (A) After a 24-hour exposure to Dioscin, HCC827 cells were subsequently incubated with MG132 (20 µM) for 8 hours. Co-immunoprecipitation (Co-IP) analysis was performed on the whole cell extract (WCE). (B) HCC827 cells were transfected with Ctrl siRNA sequence and Fbxl7 siRNA sequence for 48 h, treated with or without Diosicn for 24 h, followed by an 8-hour incubation with MG132 (20 µM), respectively. The WCE underwent analysis to study survivin ubiquitination. (C) HCC827 cells underwent transfection with Ctrl siRNA sequence and Fbxl7 siRNA sequence for 48 h, treated with or without Diosicn for 24 h, respectively, and subjected to IB analysis.

**Figure 6:**
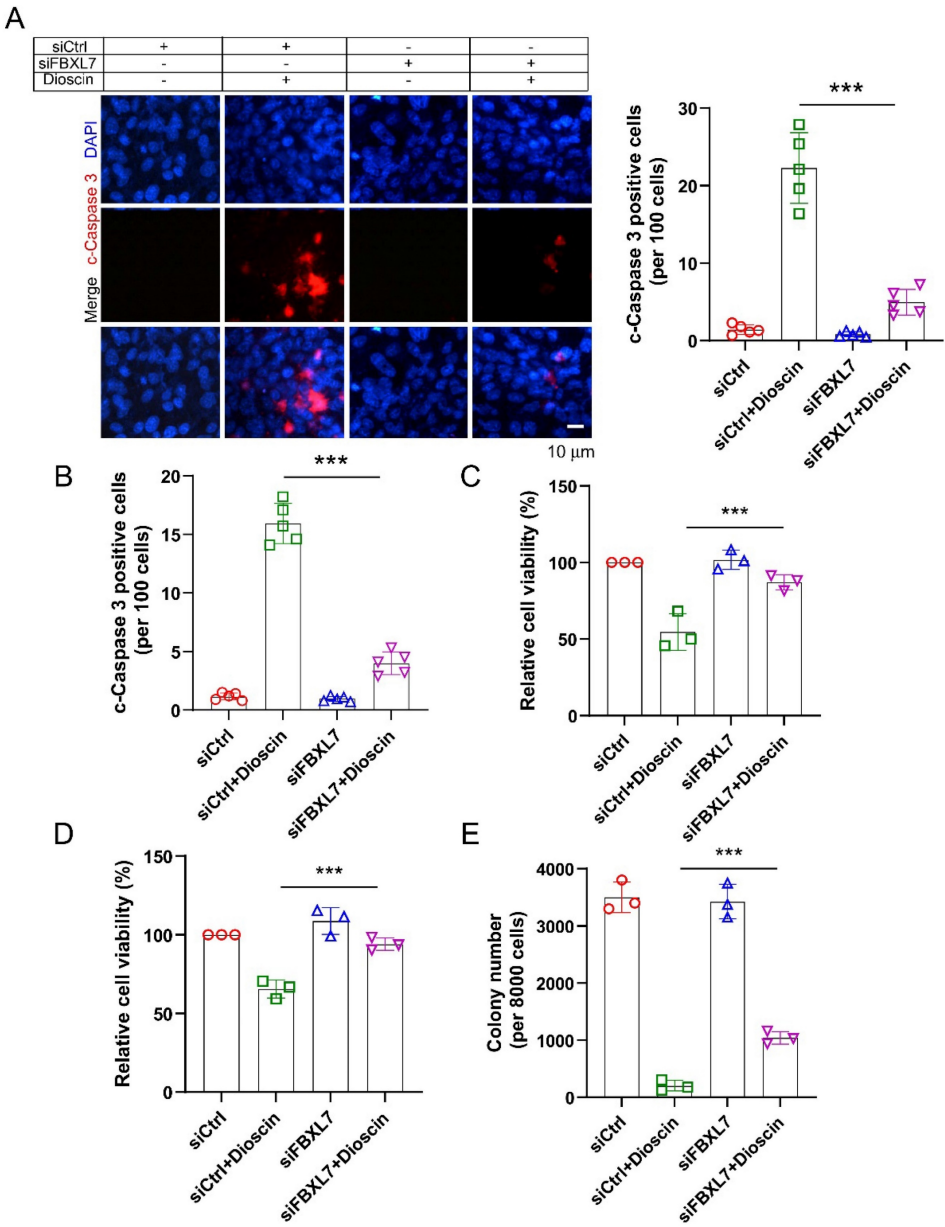
Dioscin induces NSCLC cells apoptosis in an Fbxl7-dependent manner. (A and B) Immunofluorescence (IF) assessment of c-Caspase 3 in HCC827 (A) and A549 (B) cells transfected with Ctrl siRNA sequence and Fbxl7 siRNA sequence for 48h and subsequently exposed to Dioscin treatment or left untreated for 24 hours. Scale bar, 10 μm.***p<0.001. (C-D) HCC827 (C) and A549 (D) cells transfected with ctrl siRNA sequence and Fbxl7 siRNA sequence for 48 h and subsequently exposed to Dioscin treatment or left untreated for 24 hours, cell viability was assessed using the MTS assay. (E) HCC827 cells transfected with ctrl siRNA sequence and Fbxl7 siRNA sequence for 48 h and subsequently exposed to Dioscin treatment or left untreated for 24 hours, colony formation ability by anchorage-independent cell growth assay. ***p<0.001.

Previous reports indicate that E3 ligase Fbxl7 is an essential regulator of survivin protein, regulating its expression via inducing substrate protein ubiquitination and proteasome degradation 38, 39. To investigate whether E3 ligase Fbxl7 is involved in Dioscin-induced survivin ubiquitination. Co-IP assay was conducted and the result showed that Dioscin treatment increased the interaction between Fbxl7 and survivin protein in NSCLC cells (Fig. 5A). Expectedly, knockdown Fbxl7 using siRNA impaired Dioscin-induced survivin ubiquitination in NSCLC cells, suggesting that Dioscin-induced survivin ubiquitination and degradation necessitate the presence of the E3 ligase Fbxl7 (Fig. 5B). We further explored the crucial role of E3 ligase Fbxl7 in the antitumor effect of Dioscin on NSCLC cells. Firstly, the apoptosis of cells was detected by western blotting assay. It was found that the knockdown of Fbxl7 neutralized the upregulation of c-Caspase 3 protein level induced by Dioscin (Fig. 5C). Consistently, the IF results manifested that the number of c-Caspase 3 positive cells was dramatically reduced after knockdown of Fbxl7 (Fig. 6A and B). Notably, cell viability (Fig. 6C and D) and colony formation (Fig.6E) of NSCLC cells were significantly restored in Fbxl7 knockdown cells treated with Dioscin. Overall, our data suggest that Fbxl7 is responsible for survivin degradation and apoptosis activation in Dioscin-treated NSCLC cells.

### Dioscin suppresses tumor growth of NSCLC cells *in vivo*

**Figure 7:**
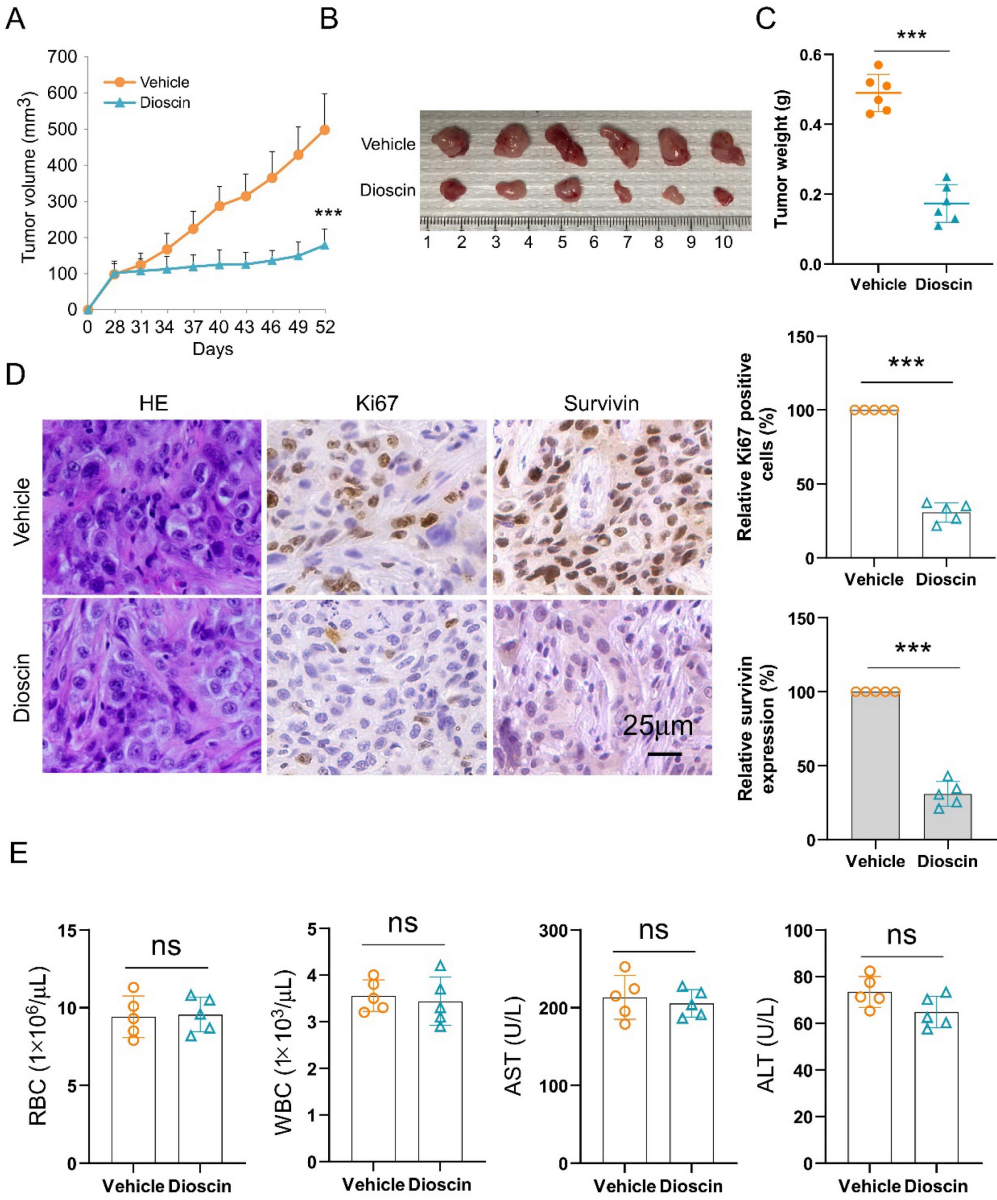
Dioscin suppresses *in vivo* tumor growth of NSCLC cells. (A-C) The tumor volume (A), The image of tumor mass (B), and tumor weight (C) in A549-derived xenograft tumors subjected to either vehicle control or Dioscin. ***p<0.001. (D) Analysis of Ki67 and survivin expression through IHC staining in A549-derived xenograft tumors. Scale bar, 25 μm. ***p<0.001. (E) Blood analysis of RBC, WBC, AST, and ALT levels in tumor-bearing mice with vehicle or Dioscin treatment. ns, not statistically significant.

To investigate the anti-tumor efficacy of Dioscin *in vivo*, xenograft mouse models were generated. The results manifested that Dioscin markedly suppressed tumor development of A549-derived xenograft tumors (Fig. 7A-7C), showing that the tumor volume (Fig. 7A and B) and tumor weight (Fig. 7C) were markedly reduced with Dioscin treatment, in which the tumor volume in the vehicle-treated group was more than 500 mm^3^, while in the Dioscin-treated group was less than 200 mm^3^ (Fig. 7A). Additionally, IHC data showed that the levels of Ki67 and survivin protein expression were significantly reduced in Dioscin-treated xenograft tumors (Fig. 7D). Furthermore, the *in vivo* tolerance of Dioscin was evaluated by blood routine analysis. It was shown that there was no significant difference in the RBC and WBC counts, AST and ALT levels between control and Dioscin-treated mice (Fig. 7E). In conclusion, these data indicate that Dioscin, a naturally occurring compound, demonstrates excellent tolerance and effectively inhibits tumor growth *in vivo* within NSCLC cells.

## Discussion

Dioscin (C_45_H_72_O_16_), a natural steroid saponin, is found in many plants and has antioxidant, anti-inflammatory and hypolipidemic, anti-obesity, hepatoprotective, and anti-tumor effects, thus protecting the body from cancer, gastrointestinal diseases, cerebrovascular diseases, and organ toxicity 40, 41. Previous studies have demonstrated that Dioscin exhibits potent antitumor effects, a property that is mainly accomplished through its regulatory effects on cell cycle, autophagy, cell migration, apoptosis, reactive oxygen radicals (ROS) generation, proliferation, Ca2^+^ release and DNA damage, as well as its mechanistic effects on relevant signaling pathways and multiple target proteins in the growth and metastasis of cancer 41-43. In addition, epithelial-mesenchymal transition (EMT) and angiogenesis, which have critical roles in tumor invasion and progression, can also be regulated by Dioscin^24^. However, its antitumor effects and potential mechanism of action on NSCLC have not been comprehensively elucidated. This study found that Dioscin significantly suppressed the malignant phenotypic properties, such as colony formation ability and cell viability. Our data identify a novel anti-tumor mechanism of Dioscin, suggesting that targeting survivin is a promising alternative strategy for treating NSCLC.

Survivin, as a crucial protumor factor in tumorigenesis, development, and poor prognosis, is an important biomarker and a promising target in tumor therapy 44, 45. Therefore, exploring the regulatory mechanism of survivin in tumor cells remains pivotal scientific research. Ubiquitin is one of the crucial ways of protein post-translational modification and is involved in mediating protein biological function and expression. At present, some ubiquitin enzymes and deubiquitinases have been proven to be closely related to the intracellular regulation of survivin^46^. Arora et al. found that E3 activity of X-linked inhibitor of apoptosis (XIAP) directly promoted survivin ubiquitination and played a critical role in XAF1-mediated survivin degradation 47. FBXL7, another E3 ubiquitin ligase, had been verified to be a survivin-interacting protein, which induced ubiquitin-proteasome degradation of survivin^38^. Consistently, in the present study, the interaction between survivin and E3 ligase Fbx17 was increased after treatment with Dioscin, which accounted for Dioscin-induced survivin ubiquitination and degradation. Several deubiquitination enzymes, such as CSN5, USP1, USP19, and STAMBPL1, prevent the ubiquitin degradation process of survivin and stabilize its expression, leading to tumor progression and therapeutic resistance 48-50. Increasingly, researchers are devoted to discovering an effective survivin-targeting agent and moving it into clinical therapy.

Summarily, there are five main categories of targeted survivin cancer therapeutics in the current: survivin immunotherapy, survivin mRNA inhibitors, survivin transcription inhibitors, survivin homodimerization and survivin-partner protein interaction inhibitors 51-53. However, developing a novel survivin-targeting agent with well-tolerance and effective antitumor activity remains challenging. YM155 is the first discovered small molecule survivin inhibitor, and it can significantly downregulate survivin expression by disrupting the survivin transcription factor Sp1, ILF3, and p54nrb in cancer cells 54-56. Plescia et al. designed a cell-permeable peptidomimetic shepherdin, which can destabilize its client protein survivin by binding to the ATP pocket of Hsp90 to induce tumor cell apoptosis 57. Natural products have attracted wide attention recently because of their low toxicity and sustainably obtained properties. A large number of preclinical studies have proved that natural compounds exert an antitumor effect by targeting a variety of pro-tumor proteins, such as survivin^58^-^60^. Xanthohumol significantly reduced survivin expression by regulating phosphorylation at Thr34 and facilitating ubiquitination, inhibiting oral squamous cell carcinoma (OSCC) cell growth 35. Dihydromyricetin, a natural product derived from ampelopsis grossedentata, exhibits profound antitumor activity against NSCLC cells by downregulating the epidermal growth factor receptor (EGFR)/Akt/survivin signal pathway 61. In the early stage of the research, we have confirmed that Dioscin exhibits obvious antitumor biological activity. Further mechanistic study illustrated that Dioscin administration remarkably activated the apoptosis signaling by facilitating the interaction between survivin and E3 ligase Fbx17 to promote survivin ubiquitination and degradation, which complements the new pharmacological activity of Dioscin in the treatment of human diseases.

## Conclusions

This study shows that the natural compound Dioscin could downregulate survivin expression to inhibit NSCLC cells by promoting Fbxl7-induced survivin ubiquitination and degradation. Our discoveries imply that Dioscin holds potential as a viable therapeutic candidate for NSCLC.
